# Reticulon 3 aggregation and its role in the formation of dystrophic neurites

**DOI:** 10.1186/1750-1326-7-S1-L23

**Published:** 2012-02-07

**Authors:** Riqiang Yan

**Affiliations:** 1 Department of Neurosciences, Lerner Research Institute, Cleveland Clinic, Cleveland, OH 44195, USA

## Background

Alzheimer’s disease (AD), the most common cause of dementia in the elderly, is characterized by the presence of Aβ plaques, which results in a progressive neuronal degeneration and subsequent cognitive decline. Dendrites and axons in the region surrounding the plaques may become damaged and swollen, forming what are known as dystrophic neurites (DNs). However, the mechanisms by which DNs affect cognitive function in AD patients are not understood.

## Methods

While characterizing the reticulon (RTN)/Nogo family of proteins, which have been shown to negatively regulate BACE1 activity, we found that RTN3, which is primarily neuronally expressed, accumulates in a distinct population of DNs called RTN3 Immunoreactive Dystrophic Neurites (RIDNs) in the brains of AD patients and in APP transgenic mice, an animal model for AD. More importantly, transgenic mice overexpressing RTN3 (Tg-RTN3) produce RIDNs in the hippocampus at a young age, while wild-type mice produce similar RIDNs only at older ages.

## Results

The importance of RIDNs in relation to cognitive decline is further evidenced by the finding that Tg-RTN3 mice where RIDNs are present show impaired learning and spatial memory, reduced spine density and synaptic plasticity. Molecular characterization of RIDNs indicates that RIDNs contain RTN3 aggregates and inducible expression of RTN3 in a mouse model reveals that RTN3 aggregates are formed irreversibly.

## Conclusion

Together, our results suggest that inhibition of RTN3 aggregation is a promising approach for improving cognitive dysfunction associated with Alzheimer’s pathology.

